# The Medical Profession: Its Nature and Tendencies

**Published:** 1886-11

**Authors:** Charles G. Stockton

**Affiliations:** Professor of Materia Medica and Therapeutics, Niagara University


THE BUFFALO Medical and Surgical Journal

Vol. XXVI. NOVEMBER, 1886. No. 4

### Original Communications.

### THE MEDICAL PROFESSION: ITS NATURE AND TENDENCIES.

By CHARLES G. STOCKTON, M. D.,

Professor of Materia Medica and Therapeutics, Niagara University.

*Mr. President, Ladies and Gentlemen:*

This evening the faculty of Niagara University has occasion to say welcome, not alone to those whose faces are familiar to these walls, but to a new class about beginning the study of a learned profession.

Nearly a hundred such gatherings as this in as many regular colleges throughout this broad land witness the congregation of ten thousand students of medicine annually.

There are already many doctors in Christendom, yet additions to the ranks are forthcoming. Upon every gala day, when young humanity crowds the thoroughfares, the eye must rest upon many future candidates for medical honors. Although they go unrecognized, nevertheless they are there, and in numbers relatively increasing.

What are the tendencies, what the social forces that thus shape the futures of so many young men? A reply introduces a broad subject, the consideration of the nature and object of the medical profession.

It is a subject that ought to be of peculiar interest to medical students, and while it can be only briefly treated in an address of this kind, there are reasons that, to my mind, justify an effort towards its accomplishment.

A man's conception of what makes up the profession can, in part, be known if he will explain his purpose for embracing it. Now, it does not appear to be an easy task for men to state their reasons for following their chosen callings in life. Indeed, they generally seem to walk into the matter heedlessly, without reaching very positive conclusions or having very definite aims, with this one remarkable exception: they go in to make money.

Probably the majority of the ten thousand fresh students purpose adopting the profession in order that they may make a living. Now, to make a living is unquestionably a most natural and honorable undertaking, and no doctor is to be blamed, I suppose, for *trying* to live by practicing medicine; but the question now before us is asked with a view of analyzing the conception that you may have as to what constitutes the profession, and the question is put to each: What is your object in entering it? Let us take the reply of the majority, and, for the sake of argument, say that you go in to make money. As a fact, this answer is rarely altogether true. A man may think his purpose is to acquire riches, but before he has been long enlisted in practice; before he has often engaged grim death in deadly conflict; before he has often felt the confiding pressure of the sick one's palm; before he is long accustomed to the sensation that his own personality brings calm and hope and cessation of pain to his fellow-creatures who entrust their lives to his judgment and skill—he will reach the conclusion that he has to do with something of graver import than the making of money.

Why, you cannot practice medicine for money. Where would it carry you; where would it carry the profession; where would it carry society?

Suppose all physicians should adopt the methods of those in trade and say: Good health has a certain commercial value; we will agree to cure your ailments for cash; a fair discount for job lots. This would have a strange effect upon the people, notwithstanding that they now have the spirit of barter so thoroughly imbued in them, that many think they would welcome such a state of affairs. And this public opinion has a great influence upon the profession, just as the profession has on public opinion. "Our deeds determine us as much as we determine our deeds."

So, suppose the profession demoralizes the people and the people demoralize the profession, until we sink to a step where we say: We are for gain; we are for ourselves; we are venal; we prepare ourselves for practice quickly, and the legislature supports us; our diplomas are of the most attractive character, and we purchase many of them; behind our names are many letters signifying many ill-gotten degrees; we advertise our medical schools for the celerity with which they pour out doctors, full-fledged, in the eye of the law. The largest class of students frequent that school having the shortest course of study and the lowest standard of examinations. Oh! yes, we are a contemptible profession, but remember that we are now venal. What care we for the race of man? It is the race for money that concerns us. We are specialists of every known organ of the human body, and we do not hesitate to say that in our researches we have reached the very *ultima thule* of knowledge, and we will have the people know it. We advertise it widely. The public speech, the public print, the public exhibition are all drawn upon. We are making a sensation.

Hold, brothers of the medical profession! Stand fast; remember the objects of our high calling, the examples of our fathers, the requirements of medical ethics. Alas, we are slipping sadly! Professional honor hampers business; so let us establish a new honor on a paying basis. Science is at a discount, and its nursing waste of time. The spirits of unnaturalized truths retrace their phantom steps into the far distance whence they came.

Does the profession seem crowded? Well, that can be arranged by creating new and artificial systems of medicine; they will compete with each other and make things interesting. Truth need not obstruct our way, since there remains no guide for the people. Establish pathies upon impossible bases, and swear to accomplish that which is impossible. Let there be vita-pathies that deal with the essence of life and trifle with the secrets of the unknowable. Water is a constituent of all organic matter; let there be hydro-pathies. Oxygen is a component part of nearly everything; let there be oxygen-pathies. That which is nothing overcomes that which is everything; let there be homœopathies. Opposition to death always defeats it; let there be allopathies. This is the age of pathies, no matter if it becomes the age of error. We have no interest, save self-interest. We are on the make.

Devote your time to learning cures and then patent them; keep them secret; sell them for all they will bring. Erect great wholesale houses of health; endow them with cheap staffs of doctors and the blind credulity of fat-headed people, and see the funds accumulate. Advertise yourselves and your houses in congress; advertise in religious papers, that were established for the sole purpose of supplying the believer with truth. Insert advertisements of pills, whose purposes are disreputable, disgraceful, inhuman and murderous, on the same page with an editorial on "the efficacy of prayer." If this doesn't fill your coffers, persuade some sort of a clergyman to certify that he was cured of the last stages of a deadly cancer that was devouring his vitals, by taking your favorite remedy. If this is insufficient, offer prizes to astronomers for catching comets. Let it become a popular impression that you are the fosterers of science and the fathers of comets.

But there are other fields. Fence in the baths, and wall round the mineral springs. Reserve the right to the operation that bears your name, publish the *good* results in the daily press and out-advertise your neighbor. Bother with the profession and with codes. The profession is a fetish, and codes are unbearable. Neither is necessary in selling groceries, nor meat, nor tin; why should they be in medicine?

Such a portrayal as this is not untruthful of the result that would follow the going of all doctors into their profession solely for personal advancement. You see that you must broaden the horizon of your purposes, else calamity will fall upon both your purposes and your profession. For, clearly, society would not support so outrageous an organization, after society had sufficiently endured. When it should be known that the profession was unworthy of confidence, then the profession would die. There must be an honest, heroic man at the bedside where one's child lies sick of diphtheria; there must be organized co-operation to direct the quarantine, and stamp out the flame of pestilence; there must be long study, slow of recognition, to enable us to understand the causes, the courses and the curses of disease.

Society needs us, and must protect us in order that she herself may be protected; and as she is elevated and pure in her regard for the profession, just in such proportion will medicine and science advance, hastening the progress of society.

Yes, this close relation between society and the profession is so necessary, that there can be little doubt that the rise of the profession was syncronous with the rise of civilization.

The early man with his powerful digestion, his unimpressionable nervous system, and his hardy thews, no more needed medicine or surgery than does the camel on the sands, than does the hibernating bear. For whether inured to the damp and cold, like the cave men, clad in the skins of beasts little more savage than themselves, or whether seasoned to the refulgence of the burning sun, driving their flocks across arid plains, from one spot of verdure to another, eating kids and drinking the milk of goats—Oh! they were not sick. Sickness! it is the price of our evolution from the life of savage tribes, into the power, beauty and usefulness of Christian nations.

Think of those remote periods, when men in rugged health slept close to their mother earth, under the cover of the night, rising each morning to wander to some new primeval habitation, far from contagion, far from the ravages of the infestuous microbe.

There were giants in those days; and but for floods and famines, but for wild beasts and bloody battles, men almost never died. They counted the centuries as years, and saw the everlasting hills grow old around them.

But with the advent of civilization came arts and artificial lives, men lived in crowds and spread disease, and then came thoughts of medicine.

When one man had more experience and success than his neighbors in relieving their complaints; when one was recognized by his community as a healer; when he accepted the situation and perceived the importance of learning and teaching—just then began the profession.

We are apt to date the rise of medicine and of the profession back to the days of Hippocrates; but, doubtless, before him for ages the world had not only her doctors but her profession of medicine. And, as a profession, contributing largely to the culture of the times, that faded as civilization faded, and brightened as civilization brightened, helping materially towards the common goal—the welfare of the race and the emancipation of man, up to the time of written history. Then came the almost divine utterances of Hippocrates. A formula of what the profession should be, came from the lips of that man with nearly the same confidence and authority as that which inspired the expounder of the ten laws graven in stone by the finger of God. So far were these utterances in advance, that the race has not even yet come to understand them, and so far ahead of the profession, that many of its members fail to apply their truth. Physicians have been too human to be Hippocratic; the practitioner too much for the individual, and not enough for the profession to bring into a state of perfection that which will yet become perfect when mankind comes into his high estate. The planet will doubtless be very gray and wrinkled ere this shall be brought about, but the time must come when the spirits of the old veterans of the regular medical profession shall assemble to celebrate the universal excellence of the cause for which they fought, perhaps, many thousand years ago.

Those of us who are there, with vision amplified, shall perceive in the retrospective that great resultant of the professional labor which now stretches away into the future, too obscure for our safe speculation. For the ends of medicine are further reaching than the accumulation of riches, or the relief of human suffering, or the immediate benefits of the culture that necessarily accompanies the study of this branch of knowledge.

There must be evolution in the profession if there is in society; and when one reaches perfection, there will it find the other. We of the nineteenth century have upon us the responsibility of helping to that end. It is a responsibility that cannot be avoided; it is a duty that we cannot go around. * * * * * * We may contrive, prevaricate and dissemble, but in truth the fact remains that the profession is not what it should be, not what it is to be; and that man who strives most to elevate and refine it, succeeds doubly, for he also elevates and refines himself. Now let me advert to the question: What is your object in studying medicine? If we agree in what has been said, the reply, it would appear, is evident. There can be but one true reply. It is, we enter medicine to improve it, and thereby to improve society and ourselves.

Is this all a nonsensical notion, a quixotic idea? No; there can be nothing more practical.

The physician who sacrifices his professional standing for selfish purposes, is a moral ruffian. He is like the vandal who knocks off the ear of a Parian figure and barters it for a shilling. He is a debaucher of medical morals, and should be punished by medical societies not only, but an acquiescence of public opinion should manifest itself, in denying to such a member the right to practice a profession which he has befouled. There should be no sympathy for such a man, except that which is accorded any other common sinner who repents, reforms and pleads for mercy. If the people and the profession would only unite in this respect, how much easier would be the struggle; which is another suggestion of the truth that the world and the profession must grow together.

It sometimes seems to a discouraged man that the task is hopeless. It seems to him that his efforts in the right direction are like the vain endeavors of Sisyphus with the stone.

This, however, is only the view of the discouraged man, and none of this kind have any business in the profession. The work before us will not be accomplished until the earth grows weary in its revolutions, and the man who tires of his small share is only a clog to the sandals of progression.

Yes, the object of every student should be as lofty as this: to improve medicine, that medicine may improve society. And society does not fail to recompense him who labors for her honestly and well. Reference is not here intended to those gifted men whose works have added millions of years of useful life to the human race; not to John Hunter, nor to Jenner, nor to Pasteur, nor to the few whose names are familiar and blessed to all, but to the every-day, well-educated, observing, thoughtful, conscientious doctor; him who does all of which he is capable for the relief of his fellow-man, who is the leader in the anabasis, and the rear guard in the katabasis of life. Society does not fail to recompense him, I say, for if he be not rich he has enough, and if he be not distinguished, he has the opportunity so to become, if his natural endowments permit of it. In medicine, the world is full of opportunities gaping to be filled, if we have but the ability to fill them. And the matter of ability is not so much what is commonly termed "natural gifts," as it is the power to work. Hard work is the talisman of success.

Gentlemen, to speak beforehand, let me ask you to put yourselves under the extraordinary influence of this talisman when you come to that department of your studies which it is my privilege to teach in this institution.

My learned and eloquent colleagues were so sensible of the importance of the subject of Materia Medica and Therapeutics, that I was se'ected to deliver this opening address, being thus afforded the first opportunity, and I take advantage of it to request that you bestow all your available time upon learning remedies and their applications.

Doubtless, you will have soon a burning affection for "the midnight oil." You will cease to indulge in current literature and fly from the blandishments of the latest novel; the theatre you will relegate to Christmas week, and the charm of the reception room will be forgotten; you will turn from politics and pleasures, and give yourselves over to a new mistress, who will prove at least as tireless as yourselves.

You will discourse upon the multitude of tonics, and puzzle your brains that, with so many of them yet, you fail to make a weak man strong. You will devote yourselves to antipyretics, and wonder how it is that as often as you down the temperature, it rises still again. You will become absorbed in the fact that there are heart stimulants which render the action of that organ strong and efficient, and yet you turn aghast when you discover that, in spite of these, the pulsations grow quicker and feebler, the respirations become irregular and shallow, the extremities turn cold and moist, the muscles relax, the face blanches, the pupils dilate, the chin falls, and, heedless of your exertions, the tortured soul slips its fetters, and is gone.

This you will see, and from it be reminded: that the therapeutist has to deal with problems that involve that mystery which we call life; that there are unknown quantities remaining in all our calculations, and you will learn that remedies are not to be arrayed in your mind as cures of maladies, and that there is not a known specific for any disease under the sun.

This philosophy is more profound than that which declares a deadly weapon for every intruder, a cure for every ail. The close interpreter of Nature renders her picture-writing into words. She says: I am mistress of the body; it develops, exists and decays in harmony with my laws; when diseased, if you would restore it to health, learn my laws and let your remedies be in compliance therewith. You may assist me, but you cannot thwart or escape me.

It would be profitable if we always thought of this before prescribing, and it would be well to learn the action of medicines with this in mind.

In army encampments, there is a "color line," and every dutiful soldier who crosses it, there salutes the flag. Now, every time that we administer drugs to a patient, we cross the Nature line, and if we are not required to doff our hats to the old dame, we have to be in other ways extremely deferential and agreeable to her, or it will be the worse for us and for our patients.

By this I mean that when we dose a man, we, in a measure, take the guidance of his vital phenomena out of the hands of Nature; and I hold that when we assume so great a responsibility, we had better remember that Nature has had a good deal more experience than we have, and the safer plan is to observe closely her methods and adhere to them. There is no department in medicine where a man requires so much knowledge as in Materia Medica and Therapeutics, in order that he may be turned loose upon a suffering world as a young doctor—safely.

May you so learn this subject, that when you come to apply your knowledge at the bedside, it will be with this rule in mind: If you don't *know* what to give, give nothing.

